# The moderating effect of cultural competence educational needs on the relationship between transcultural self-efficacy and cultural competence in Korean public health nurses

**DOI:** 10.1186/s12912-023-01253-5

**Published:** 2023-04-12

**Authors:** Young-Ran Han, Yeo-Won Jeong

**Affiliations:** 1 Department of Nursing, College of Nursing, Dongguk University, 123 Dongdae-ro, Gyeongju-si, Gyeongsangbuk-do 38066 Republic of Korea; 2 Department of Nursing, College of Nursing, Dongguk University, 123 Dongdae-ro, Gyeongju-si, Gyeongsangbuk-do 38066 Republic of Korea

**Keywords:** Cultural diversity, Nurses, Public health, Self-efficacy, Culturally competent care, Educational needs Assessment

## Abstract

**Background:**

In an increasingly multicultural society, cultural competence and transcultural self-efficacy of public health nurses is important for providing culturally congruent care for client from diverse cultural background. To improv this, it is needed tailored and effective educational program based on the cultural competence educational needs. This study investigated the moderating effect of cultural competence educational needs on the relationship between transcultural self-efficacy and cultural competence.

**Methods:**

This cross-sectional study recruited 217 public health nurses in Korea using convenience sampling from August 2018 to January 2019. A direct questionnaire was used to collect data. Study variables were analyzed using descriptive statistics, correlation, and the Hayes PROCESS macro (Model 1) moderation model.

**Results:**

The mean scores for transcultural self-efficacy, cultural competence educational needs, and cultural competence were 62.33 ± 11.08, 58.19 ± 15.08, and 97.96 ± 17.09, respectively. Transcultural self-efficacy and cultural competence educational needs were positively associated with cultural competence. In the tested model, cultural competence educational needs had a conditional moderating effect on the relationship between transcultural self-efficacy and cultural competence. The positive association between transcultural self-efficacy and cultural competence was significant at low, medium, and high levels of cultural competence educational needs and stronger for those with high needs.

**Conclusions:**

Cultural competence educational needs may be an important determinant of cultural competence among public health nurses. To effectively increase cultural competence, transcultural self-efficacy should be increased by education programs tailored by cultural competence educational needs.

## Backgroud

In 2020, foreign residents, such as migrant women who married locals, foreign workers, and international students constituted 4.1% (2,146,748 people) of the total population of Korea, which is approximately four times higher than the 1.1% measured in 2006 [1]; the multicultural nature of the society is accelerating. Amid this, foreign residents face various stresses in migrating and adapting to an unfamiliar environment, and look for medical institutions for pregnancy and childbirth, health management, and disease. Foreign workers also seek medical institutions for health risks due to poor working conditions [2]. The increase in cultural diversity in the community challenges the healthcare system to provide high-quality care to clients with diverse cultural backgrounds and needs [3]; therefore, efforts are being made to develop health polices and services based on cultural diversity [4].

Public health nurses in public health institutions, such as public health centers, are on the frontline of provision of public health services focusing on cultural diversity to meet the needs of multicultural clients; therefore, cultural competence is necessary [5]. Cultural competence is the ability to provide effective, safe, and quality care to individuals from diverse cultural backgrounds, and is a dynamic and evolutionary process considering the diverse aspects of their cultures [6]. Improving nurses’ cultural competence will reduce medical inequality in the planning of care and related activities according to the patient’s cultural background, laying the foundation for holistic care. Through this, patient satisfaction and confidence in the health services, and quality of life, is improved, and public health can be further improved [3, 6]. In an increasingly multicultural society, the cultural competence of public health nurses is essential for culturally congruent care, which is the goal of transcultural nursing [3].

Transcultural nursing skills needed to assess, plan, implement, and evaluate culturally-congruent care have cognitive, affective, and practical dimensions [7]. Cultural competence is a multidimensional learning process that integrates transcultural nursing skills in three dimensions; cognitive, affective, and practical, and transcultural self-efficacy is a major influencing factor. [7, 8]. Transcultural self-efficacy is perceived confidence, that can learn and use transcultural nursing skills to provide culturally congruent care to clients from diverse cultural backgrounds [8], and is a crucial component of cultural competence [7]. Nurses with high transcultural self-efficacy have high confidence that they can overcome obstacles in performing transcultural nursing, and furthermore, are highly motivated and actively seek help to learn transcultural nursing skills and develop cultural competence, and as a result, provide care with high levels of cultural competence [7]. In contrast, nurses with low transcultural self-efficacy may give up and avoid cultural assessments without even trying, which may directly negatively affect the development of cultural competence [7].

Since culturally-congruent care is affected by transcultural self-efficacy perceptions and educational exposure to transcultural nursing concepts and skills, nursing students and nurses need formalized education to meet diverse individual cultural care needs [8]. To tailor cultural competence education and effectiveness for the target group, it is important to understand their educational needs [9]. In Kim’s study [10] on cultural competence educational needs, hospital nurses scored higher on attitude, skill, and cultural communication than on knowledge of theory and research and knowledge of key concepts. Lee, et al., [11] also found that nursing students’ cultural competence educational needs showed high attitude and skill for cultural communication. However, to the best of our knowledge, there are no studies on the cultural competence educational needs of public health nurses [12].

Additionally, looking at previous studies on cultural competence, transcultural self-efficacy, and cultural competence educational needs, transcultural self-efficacy is significantly associated with cultural competence [13] and positively correlated with cultural competence educational needs [10]. Moreover, cultural competence is also significantly associated with cultural competence educational needs [11]. In other words, in the relationship between transcultural self-efficacy and cultural competence, cultural competence educational needs, which is an influencing factor for both, is expected to have a moderating effect, however, there are no studies related this.

Cultural competence and transcultural self-efficacy are essential for public health nurses to provide holistic care to multicultural clients in transcultural nursing. Cultural competence can be increased through education [12]. Therefore, to provide customized and efficient cultural competence education, considering the characteristics of public health nurses, it is important to understand their cultural competence educational needs, and examine the level of cultural competence educational needs, cultural competence, and transcultural self-efficacy of public health nurses, and the relationship between them as a whole.

**Fig. 1:**
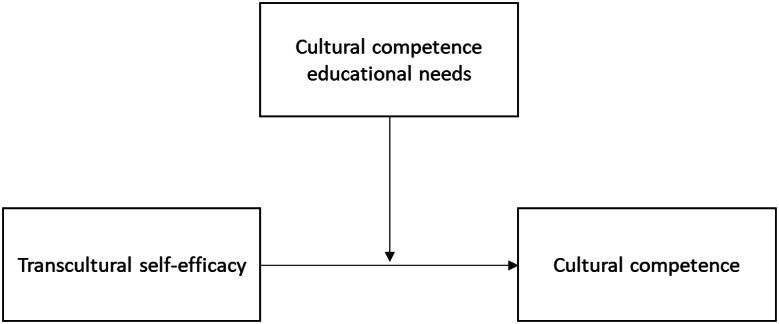
Hypothesis model of the study

Therefore, this study aimed to assess the levels of transcultural self-efficacy and cultural competence, and cultural competence educational needs, of public health nurses in Korea. In addition, the moderating effect of the cultural competence educational needs on the relationship between transcultural self-efficacy and cultural competence was evaluated. The proposed hypotheses were as follows (Fig. 1):

- Hypothesis 1 (H1): Transcultural self-efficacy would be positively associated with cultural competence.
- Hypothesis 2 (H2): Cultural competence educational needs would have a moderating effect between transcultural self-efficacy and cultural competence.

## Methods

### Study Design and aim

A cross-sectional study design was used to evaluate the moderating effect of cultural competence educational needs between the transcultural self-efficacy and cultural competence of public health nurses in Korea.

### Setting and Participants

In Korea, various national policies, such as health examination, vaccinations, health information provision and counseling for multicultural families are being implemented at public health centers, that are mainly handled by public health nurses [14]. Additionally, public health nurses and other nurses in Korea, are required to receive at least 8 h of continuing education per year from the public health nurses association or hospitals. In this study, participants were recruited from public health nurses who attended a continuing education held by public health nurses association for public health nurses working in public health centers in four metropolitan cities and four provinces, using a convenience sampling method.

The inclusion criteria for participants were public health nurses: (1) working at public health centers in Korea; (2) having more than one month’s experience at public health centers; (3) who understood the study’s purpose and voluntarily agreed to participate in the study. For regression analysis, the sample size was calculated using the G*Power 3.1.9.2 program. The minimum number of participants needed for statistical power of 0.95 at a significance level of 0.05, and 12 predictors based on an effect size of 0.15, was 184. Considering an expected dropout rate of 20%, a total of 220 participants were recruited, and 217 valid questionnaires were used in the final analysis.

### Measurements

#### 1) Transcultural self-efficacy

Transcultural self-efficacy was measured using the Korean version of transcultural self-efficacy scale, considering the transcultural context of Korea, developed by Oh et al. [4] based on the model of cultural competence and confidence [8]. The scale consisted of 25 items with three subscales. The cognitive domain (4 items) measured the nurses’ assurance in recognizing the difference between the cultural characteristics of individuals with different cultural backgrounds and the nurse’s own cultural characteristics. The practical domain (12 items) measured the nurses’ assurance in effective practical activities for individuals from different cultural backgrounds, and the affective domain (9 items) measured the nurses’ assurance in understanding, accepting, and respecting individuals from different cultural backgrounds. Each item was evaluated with a four-point Likert scale (1 = not sure at all, 4 = very sure). The scores ranged from 25 to 100 with higher scores implying higher transcultural self-efficacy. The scale’s construct validity has been confirmed using exploratory factor analysis, concurrent validity, convergent and discriminative validity, and criterion validity [4]. Additionally, good internal consistency of the scale has been reported; Cronbach’s ⍺ was 0.88 in Oh et al.‘s study [4]; it was 0.945 in this study.

#### 2) Cultural competence educational needs

To evaluate cultural competence educational needs, the modified Korean version of the blueprint for integration of cultural competence in the curriculum questionnaire (BICCCQ) was used [10]. Kim [10] translated and validated the Korean version of BICCCQ, which was developed by Tulman and Watts [15]. The Korean version of BICCCQ consists of 36 items, with attitude and skill (11 items), knowledge (21 items; basic nine items,; theory and research seven items; key concepts 5five items), and cultural communication (four items). To focus on the practical field of public health, the authors modified the Korean version of BICCCQ to 25 items with attitude and skill (10 items), knowledge (12 items; basic seven items; theory and research one item; key concepts four items), and cultural communication (3 items). Each item was evaluated with a five-point Likert scale (1 = not necessary at all, 5 = very necessary). The total scores ranged from 25 to 125. In addition, the average score was calculated, and higher scores suggested higher cultural competence educational needs. The tool’s reliability at the time of development was measured using Cronbach’s ⍺ = 0.960 [15]. In addition, the Korean version of the BICCCQ has confirmed the content validity and good internal consistency;[15]; Cronbach’s ⍺ = 0.990 [10]. In this study, Cronbach’s ⍺ was 0.974.

#### 3) Cultural competence

To assess participants’ cultural competence, the Korean version of short form of the cultural competence scale developed by Chae and Park [16] for Korean nurses was used. This scale consisted of 14 items with four subscales: cultural awareness (4 items), cultural knowledge (3 items), cultural sensitivity (4 items), and cultural skills (3 items). Each item was ranked using a seven-point Likert scale (1 = strongly disagree, 7 = strongly agree), and the total score ranged from 14 to 98 with higher scores suggesting higher cultural competence. The scale demonstrated good validity through confirmatory factor analysis, convergent validity, discriminant validity, known-group comparisons [16]. It has also been reported to have good internal consistency; Cronbach’s ⍺ was 0.89 [16], and in this study, it was 0.931.

#### 4) Covariates

The covariates included age, sex, marital status, education, nurse specialist certification, and the duration of work experience in public health centers. Additionally, experience of cultural diversity was assessed by the question “Have you ever had the experience of residing abroad for at least a month, foreign travel, or foreign training programs?” (yes or no). Foreign language proficiency level was assessed by the question “Are you fluent in any foreign languages, including English?” which was evaluated with a four-point Likert scale (1 = not fluent at all, 2 = a little, 3 = moderate, 4 = very fluent). Experience of caring for multicultural clients was assessed by the question “Have you ever cared for multicultural clients?” (yes or no) and “If yes, please describe the type of client and the type of care.” Lastly, education on multicultural nursing was assessed by the question “Have you ever had education on multicultural nursing?” and “If yes, please describe the type of education, such as classes in university or job training, etc.”

### Data collection procedures

Data were collected from August 2018 to January 2019. First, we obtained permission for the study from the public health nurses association which is in charge of continuing education for public health nurses. Thereafter, one of the researchers contacted the manager in charge of continuing education at the public health nurses association and explained the study’s purpose, procedure, and questionnaire content. In addition, everyone was informed that participation was voluntary, and that participants could withdraw at any time during the study without any negative consequences. Before the continuing education session began, the manager of the public health nurses association explained the research-related information to the public health nurses, and questionnaires in Korean were distributed. The completed questionnaires were collected at the end of the continuing education session. Of the 220 print-based questionnaires with consent forms distributed by the manager to public health nurses who voluntarily agreed to participate, all were returned with written consent. Finally, 217 valid questionnaires, excluding three questionnaires with missing data, were used for data analysis. All data were kept confidential.

### Data analysis

The data were analyzed using SPSS/WIN 25.0 (IBM Corp, Armonk, NY, USA) and SPSS PROCESS macro, version 3.4. The general characteristics and main variables were analyzed using descriptive statistics. Correlations with the main variables were processed using Pearson’s correlation coefficient. To test the study’s hypothesis, the PROCESS macro for SPSS (model 1) [17] was used. In addition, the Johnson–Neyman and pick-a-point methods were used to examine the moderating effect of cultural competence educational needs between transcultural self-efficacy and cultural competence. The regions of significance yielded statistical significance transitions within the observed range of moderators using the Johnson–Neyman method. The pick-a-point method was used to plot conditional effects for low (mean-1 × SD), medium, and high (mean + 1 × SD) levels of cultural competence educational needs. The significance of the conditional effect of the cultural competence educational needs was identified with p < .05 and when the confidence interval (CI) did not include zero [18].

## Results

### Description of the study participants and variables

The general characteristics of the study participants are shown in Table 1. All the participants were women with a mean age of 44.30 ± 10.39 years. Of the 217 participants, 20.3% (44) had one or more nurse specialist certification(s) and the mean work experience in public health centers was 12.28 ± 10.54 years. Approximately 59.4% (129) had experience of cultural diversity, such as residing abroad for at least one month, a foreign training program, or traveling. The ability to speak English as a second language, other than Korean, was low, with an average of 1.47 ± 0.52. Of all the participants, 52.1% (113) had experience with caring for multicultural clients, with the main clients being multicultural families or foreign workers. The main areas of care were health check-ups, first aid, vaccination, and visiting client’s homes to provide nursing care. A total of 18.0% (39) had received education on multicultural nursing at college or university, graduate school, or job training at public health centers.

**Table 1:** General characteristics of the study participants (N = 217)

Variables	N (%) or Mean ± SD	Range
Age (years)	44.30 ± 10.39	27 ~ 67
Sex		
Male	0	
Female	217 (100)	
Marital status		
Single	60 (27.6)	
Married	154 (71.0)	
Divorced	3 (1.4)	
Education degree		
Associate degree	62 (28.6)	
Bachelor’s degree	127 (58.5)	
Master’s degree or higher	28 (12.9)	
Nurse specialist certification		
Yes	44 (20.3)	
No	173 (79.7)	
Working experience in public health centers (years)	12.28 ± 10.54	0.17 ~ 33.5
Experience of cultural diversity*		
Yes	129 (59.4)	
No	88 (40.6)	
Foreign language proficiency level	1.47 ± 0.52	1 ~ 3
Experience of caring for multicultural clients		
Yes	113(52.1)	
No	104(47.9)	
Education on multicultural nursing		
Yes	39(18.0)	
No	178(82.0)	

*Cultural diversity included residence in abroad at least a month, foreign training program, or traveling.

The mean scores for transcultural self-efficacy, cultural competence educational needs, and cultural competence were 62.33 ± 11.08, 97.96 ± 17.09, and 58.19 ± 15.08, respectively (Table 2). The Mean item scores of each variable were transcultural self-efficacy 2.49 ± 0.44, cultural competence educational needs 3.92 ± 0.68, and cultural competence 4.16 ± 1.08. In case of cultural competence, the mean item score, 4.16 was moderate at 59.4% of the highest possible score of 7. Transcultural self-efficacy, 2.49 was also moderate at 62.3% of the highest possible score of 4. Cultural competence educational needs(3.92) was moderate to high at 78.6% of the highest possible score of 5. Regarding subscale of cultural competence educational needs, knowledge of key concepts was highest at 3.97 ± 0.79, followed by cultural communication (3.96 ± 0.73), attitudes and skills (3.93 ± 0.73), basic knowledge (3.88 ± 0.71), and knowledge of theory and research (3.76 ± 0.91).

**Table 2:** Descriptive statistics of the main variables

Variables	Domain score		Mean item score of domain*	
	Mean ± SD	Range	Mean ± SD	Range
Transcultural self-efficacy (total)	62.33 ± 11.08	26–98	2.49 ± 0.44	1-3.92
Cognitive dimension	8.65 ± 2.16	4–16	2.16 ± 0.54	1–4
Practical dimension	27.85 ± 6.41	12–48	2.32 ± 0.53	1–4
Affective dimension	25.84 ± 4.65	9–36	2.87 ± 0.51	1–4
Cultural competence educational needs (total)	97.96 ± 17.09	25 ~ 125	3.92 ± 0.68	1–5
Attitudes and skills	39.30 ± 7.29	10–50	3.93 ± 0.73	1–5
Knowledge	46.80 ± 8.62	12–60	3.90 ± 0.72	1–5
Basic knowledge	27.13 ± 5.00	7–35	3.88 ± 0.71	1–5
Knowledge of theory and research	3.76 ± 0.91	1–5	3.76 ± 0.91	1–5
Knowledge of key concepts	15.90 ± 3.17	4–20	3.97 ± 0.79	1–5
Cultural communication	11.87 ± 2.19	3–15	3.96 ± 0.73	1–5
Cultural competence (total)	58.19 ± 15.08	14–92	4.16 ± 1.08	1-6.57
Cultural awareness	18.32 ± 5.45	4–28	4.58 ± 1.36	1–7
Cultural knowledge	10.18 ± 3.81	3–20	3.39 ± 1.27	1-6.67
Cultural sensitivity	17.60 ± 5.50	4–28	4.40 ± 1.37	1–7
Cultural skill	12.09 ± 3.73	3–21	4.03 ± 1.24	1–7

*Mean item score for the domain was calculated by dividing the sum of the domain items by the number of domain items.

### Correlations among study variables

As shown in Table 3, cultural competence was positively correlated with transcultural self-efficacy (r = .357, *p* < .001) and cultural competence educational needs (r = .326, *p* < .001). However, transcultural self-efficacy was not significantly correlated with cultural competence educational needs (r = .089, *p* = .193).

**Table 3:** Correlation among study variables

Variables	TSE	CD	PD	AD	CCEN	ASK	K	CCM	CC	CA	CK	CS	CSK
TSE	1												
CD	0.669^**^	1											
PD	0.928^**^	0.611^**^	1										
AD	0.793^**^	0.287^**^	0.550^**^	1									
CCEN	0.089	− 0.049	0.028	0.196^**^	1								
ASK	0.057	− 0.042	0.008	0.146^*^	0.945^**^	1							
K	0.094	− 0.055	0.032	0.205^**^	0.959^**^	0.820^**^	1						
CCM	0.133	− 0.025	0.067	0.237^**^	0.887^**^	0.815^**^	0.816^**^	1					
CC	0.357^**^	0.218^**^	0.357^**^	0.258^**^	0.326^**^	0.329^**^	0.287^**^	0.322^**^	1				
CA	0.243^**^	0.095	0.252^**^	0.187^**^	0.331^**^	0.322^**^	0.302^**^	0.322^**^	0.868^**^	1			
CK	0.316^**^	0.329^**^	0.303^**^	0.183^**^	0.164^*^	0.199^**^	0.118	0.149^*^	0.754^**^	0.549^**^	1		
CS	0.248^**^	0.089	0.245^**^	0.212^**^	0.308^**^	0.292^**^	0.289^**^	0.299^**^	0.818^**^	0.586^**^	0.459^**^	1	
CSK	0.401^**^	0.277^**^	0.404^**^	0.271^**^	0.214^**^	0.226^**^	0.173^*^	0.237^**^	0.798^**^	0.625^**^	0.549^**^	0.510^**^	1

Abbreviation: (1) *TSE* Transcultural self-efficacy, *CD* cognitive dimension, *PD* practical dimension, *AD* affective dimension (2) *CCEN* cultural competence educational needs, *ASK* attitudes and skills, *K* knowledge, *CCM* cultural communication (3) *CC* cultural competence, *CA* cultural awareness, *CK* cultural knowledge, *CS* cultural sensitivity, *CSK* cultural skill

^*^p < .05 ^**^p < .01

### Moderating effect of cultural competence educational needs

Model 1 of PROCESS macro was used the test hypothesis 2 in this study [17]. We controlled for all covariates in the moderating analysis. Table 4 shows the moderating effect of cultural competence educational needs. Transcultural self-efficacy had a significant positive effect on cultural competence (B = 0.420, p < .001), and study hypothesis 1 was supported. Cultural competence educational needs moderated the relationship between transcultural self-efficacy and cultural competence (B = 0.009, p = .016). In other words, the higher the cultural competence educational needs, the higher the transcultural self-efficacy on cultural competence. Study hypothesis 2 was supported.

**Table 4:** Impact of transcultural self-efficacy on cultural competence and the moderating effect of cultural competence educational needs

Variables	Cultural competence				
	B	SE	*P*	LLCI	ULCI
Constant	62.372	12.382	< 0.001	37.956	86.787
Transcultural self-efficacy	0.420	0.090	< 0.001	0.243	0.598
Cultural competence educational needs	0.267	0.055	< 0.001	0.158	0.376
TSE X CC educational needs	0.009	0.003	0.016	0.001	0.016
F	5.363				
R^2^	0.520		*p* < .001		
△R^2^	0.021		*P* = .016		

Note: Values were controlled for covariates (all general characteristics).

Abbreviations: TSE, transcultural self-efficacy; CC, cultural competence; △R^2^, R^2^ change due to interaction term; SE, standard error; LLCI, lower-level confidence interval; ULCI, upper-level confidence interval.

**Fig. 2:**
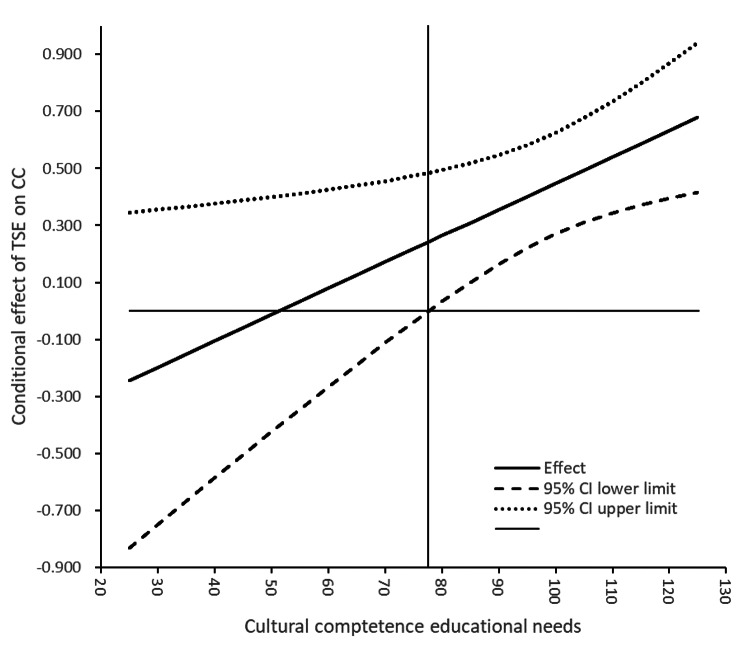
The conditional effect of transcultural self-efficacy on cultural competence at the values of cultural competence educational needs

The moderating effect of cultural competence educational needs results from the Johnson–Neyman method indicated no significant effect for cultural competence educational needs scores ≤ 77.609(10.14%). The pick-a-point method (Table 5) indicated that the positive association between transcultural self-efficacy and cultural competence was significant at relatively low (β = 0.263, SE = 0.113, 95% CI 0.039, 0.488), moderate (β = 0.420, SE = 0.090, 95% CI 0.243, 0.598), and relatively high levels (β = 0.577, SE = 0.108, 95% CI 0.364, 0.791) of cultural competence educational needs. Therefore, the positive effect of transcultural self-efficacy on cultural competence increased as cultural competence educational needs increased. The slope in Fig. 2 reflects that the significant conditional effects of transcultural self-efficacy on cultural competence was found when cultural competence educational needs was above 77.609.

**Table 5:** Conditional effects of transcultural self-efficacy on cultural competence at different levels of cultural competence educational needs

Value of cultural competence educational needs	Effect	SE	t	P	LLCI	ULCI
Mean-1 × SD	0.263	0.113	2.316	0.021	0.039	0.488
Mean	0.420	0.090	4.673	< 0.001	0.243	0.598
Mean + 1 × SD	0.577	0.108	5.336	< 0.001	0.364	0.791

Abbreviations: SD, standard deviation; SE, standard error; LLCI, lower-level confidence interval; ULCI, upper-level confidence interval.

## Discussion

To the best of our knowledge, this study is the first study to explore the moderating effect of cultural competence educational needs on the relationship between transcultural self-efficacy and cultural competence. The result indicated that public health nurses with high transcultural self-efficacy and high cultural competence educational needs had greater cultural competence. Additonally, cultural competence educational needs moderated the association between transcultural self-efficacy and cultural competence.

In this study, the cultural competence of public health nurses in Korea was 4.16 out of 7. Because there is no common standard for cultural competence, this study compared the percentage derived by dividing the mean score by the highest possible score in the cultural competence tool used in previous studies. For example, the cultural competence of visiting nurses in Korea, measured on the Korean version of the cultural awareness scale, was 61.8% (3.09 out of 5) in the study by Suk et al. [19]. Additionally, the cultural competence of clinical nurses in Korea, measured on the healthcare scale, was 53.8% (2.69 out of 5) [20], similar to the result of our study (59.4%). However, the cultural competence of public health nurses in Korea was lower than that in other countries. In Cai et al.‘s study [9], nurses working in hospitals in China scored 70.1% (101.7/145) on the cultural competence inventory for nurses, and in Lin, Mastel-Smith et al’s. study [21], Taiwanese nurses scored 67.1% (109.99/164) on the nurses’ cultural competence scale. Additionally, in Almutairi et al.‘s study [22], Canadian nurses scored 74.6% (5.22 out of 7) on critical cultural competences. These results can be considered in two ways. First, the first five-year foreign policy plan was deliberated and approved in 2008, when the era of one million foreigners was reached in Korea. In addition, multicultural studies in nursing started in 2006 [23]. Therefore, the period of full-fledged interest in multiculturalism was shorter than that in other countries. Second, the average age of our study participants was 44 years. Multicultural-related subjects were not part of the regular curriculum when they were undergraduates. Accordingly, the absence of basic education related to multiculturalism can be considered. The cultural competence of nursing students in Korea who are currently receiving multicultural nursing courses in the regular nursing curriculum is about 71.1% [13], which is about 13% higher than that of the current public health nurses. Therefore, it is essential not only to provide basic education related to multiculturalism in undergraduate education, but also to recommend training, which is fit their educational needs considering social contact, for current public health nurses who did not receive this training as undergraduates.

The transcultural self-efficacy of Korean public health nurses was moderate and consistent with a previous study for hospital nurses, which used the same tool [24]. However, it was higher than the 45.4% (4.54 out of 10) reported in the study by Kim [10]. In addition, hospital nurses had little experience in caring for multicultural patients [10], however, 52.1% of the participants in this study had experience in nursing multicultural patients. Therefore, it can be interpreted that the multicultural nursing experience of public health nurses increased as Korea gradually entered a multicultural society, which improved confidence in using cultural nursing techniques with patients. In other words, it is consistent with the results of previous studies that increased clinical encounters with multicultural patients increase transcultural self-efficacy [10, 25].

Along with this, the cultural competence educational needs of Korean public health nurses was 78.4%, which was higher than that in previous studies [10, 20][10][20]However, only 18% of the participants of this study received multicultural education in contrast to such high educational needs and transcultural self-efficacy. In contrast, current public health nurses showed the highest cultural competence-related knowledge on key concepts in the cultural competence educational needs in this study, unlike the low knowledge on key concepts in previous studies [10, 11, 20]. This may be because the participants of the previous studies were relatively young (29–31 years) or nursing school students who completed multicultural-related subjects in the regular curriculum. According to the results of this study, the educational needs for transcultural self-efficacy and cultural competence are also moderate to high as public health nurses experience of nursing multicultural patients in Korea increases. However, their experience of cultural education was minimal. Therefore, when developing cultural education for current public health nurses, it is necessary to address the fundamental concepts of culture and cultural nursing as important. In addition, institutional support is needed to increase the understanding of multicultural nursing and expand and strengthen continuing education that can be applied to practice.

In this study, cultural competence educational needs showed a positive moderating effect between transcultural self-efficacy and cultural competence. In addition, it was confirmed that cultural competence educational needs increased, the influence of transcultural self-efficacy on cultural competence increased. Therefore, cultural competence educational needs are an important factor in the cultural competence and transcultural self-efficacy of public health nurses, which can be explained by several factors, as follows. Transcultural self-efficacy is based on the concept of self-efficacy proposed by Bandura [8, 26]. Self-efficacy (confidence) is an important factor influencing the motivation for developing cultural competence [27], while also being a factor that positively affects cultural competence [13]. Motivation can be best induced when interest, or attention to content, precede learning [27]. Interest or attention to content is linked to educational needs, and the higher the level, the higher the motivation, which leads to self-efficacy, learning, and achievement [28]. In other words, when the educational needs and level of interest in cultural competence of public health nurses increase, it can potentially increase motivation for developing and improving cultural competence, which is the achievement level. In previous studies, cultural competence educational needs were also reported as factors affecting cultural competence [11]. In addition, transcultural self-efficacy was significantly related to the cultural competence of public health nurses with low, medium, and high levels of cultural competence educational needs. Moreover, the association of transcultural self-efficacy and cultural competence was stronger among those who had a high level of cultural competence educational needs in this study. In other words, the higher the cultural competence educational needs, the higher the level of cultural competence increases as the confidence that cultural nursing skills can be performed for various patients increases. Therefore, it is necessary to address the cultural competence educational needs to improve the cultural competence of public health nurses. Based on previous studies [10, 29], continuous education on multiculturalism or exposure to information through media or newsletters can be considered to meet the educational needs of public health nurses for cultural competence. In addition, it is necessary to promote transcultural self-efficacy to improve cultural competence, which can be enhanced through cultural education [30]. Based on the results of this study, rather than simply providing cultural education, it is better to identify the cultural competence educational needs of public health nurses in advance and develop and apply tailored cultural education based on this to improve the transcultural self-efficacy of public health nurses. This can facilitate improvement in cultural competence. Finally, we suggest future studies that identify the influence of the motivations related to the development of cultural competence on transcultural self-efficacy and cultural competence, which were not directly addressed in this study.

The limitations of this study are as follows. First, the study used convenience sampling, and all participants in this study were women. Therefore, it is not representative of all public health nurses in Korea. Future research should test this using more representative samples including male. [7, 9, 10, 13] Second, this was a cross-sectional study, which limits the interpretation of causality. Hence, future research can be improved through longitudinal studies. Third, numerous factors influenced cultural competence, and in this study, only cultural competence educational needs was examined as a moderating variable. Transcultural self-efficacy and cultural competence educational needs could explain a limited portion of cultural competence. Therefore, it is recommended that further research explore the various influencing factors that affect cultural competence. [9, 13, 27]. Finally, there may be a social desirability bias because this study is based on self-reported data. Therefore, caution should be applied in the interpretation of the results of this study.

## Conclusion

In Korea, compared to clinical nurses, public health nurses have more opportunities to meet and care for multicultural families, [19]therefore, the cultural competence of public health nurses is essential for culturally congruent care. The results of this study showed that the cultural competence of public health nurses in Korea was moderate, however it was lower than that in other countries. Cultural competence educational needs and transcultural self-efficacy were moderate to high, but only 18% of those received cultural education. Therefore, cultural education is necessary for them. Cultural competence educational needs showed the moderating effect of a positive relationship between transcultural self-efficacy and cultural competence. In addition, the higher the cultural competence educational needs, the stronger the relationship between transcultural self-efficacy and cultural competence. Therefore, when applying cultural competence and transcultural self-efficacy education targeting current public health nurses, it is necessary to identify the cultural competence educational needs in advance and develop a tailored education program targeting them. In addition, when developing cultural education, it is necessary to focus on the main concepts of culture and cultural nursing. Moreover, institutional support is needed to enhance understanding of multicultural nursing and expand and strengthen continuing education applicable to practice, enabling public health nurses to provide transcultural nursing.

## Data Availability

The datasets analyzed during the current study are not publicly available due to privacy or ethical restrictions but are available from the corresponding author on reasonable request.
